# *Listeria monocytogenes* Meningitis in Adults: The Czech Republic Experience

**DOI:** 10.1155/2013/846186

**Published:** 2013-09-11

**Authors:** Olga Dzupova, Hanus Rozsypal, Dita Smiskova, Jiri Benes

**Affiliations:** ^1^Charles University in Prague, Third Faculty of Medicine, Department of Infectious Diseases, Na Bulovce Hospital, Budinova 2, 180 81 Prague, Czech Republic; ^2^Charles University in Prague, First Faculty of Medicine, Department of Infectious Diseases, Na Bulovce Hospital, Budinova 2, 180 81 Prague, Czech Republic; ^3^Charles University in Prague, Second Faculty of Medicine, Department of Infectious Diseases, Na Bulovce Hospital, Budinova 2, 180 81 Prague, Czech Republic

## Abstract

*Background*. *Listeria monocytogenes* (LM) is currently the third most frequent pathogen of bacterial meningitis in adults. *Methods*. A prospective study of patients with LM meningitis in a Czech tertiary care hospital, carried out from 1997 to 2012. *Results*. Thirty-one patients were diagnosed with LM meningitis, which was 7% of a total of 440 adult patients with acute bacterial meningitis (ABM) over a 16-year period. Their median age was 63 years, range 26–80 years. Nineteen patients (61%) had underlying immunocompromising comorbidity; 15 patients (48%) were older than 65 years. Fourteen patients (45%) had arterial hypertension. The typical triad of fever, neck stiffness, and altered mental status was present in 21 patients (68%). The median count of cerebrospinal fluid (CSF) leukocytes was 680/*μ*L, protein level 2.6 g/L, and glucose ratio 0.28. Four patients (13%) died, and nine (29%) survived with moderate to severe sequelae. *Conclusion*. LM meningitis is known to affect immunosuppressed and elderly patients. Arterial hypertension seems to be another important predisposing factor. Clinical symptoms, CSF findings, and disease outcomes, did not significantly differ from other community-acquired ABM in our study, although the CSF leukocyte count was lower. Ampicillin showed good clinical and bacteriological efficacy in the majority of patients.

## 1. Introduction

*Listeria monocytogenes* (LM) meningitis and other central nervous system (CNS) manifestations occur rather sporadically and primarily affect predisposed individuals. The number of human cases is supposed to rise up both absolutely due to increasing population of immunocompromised and elderly persons and relatively due to decreasing incidence of meningitis caused by *Haemophilus influenzae*, *Streptococcus pneumoniae*, and* Neisseria meningitidis* in the era of vaccination. As a result, LM has become the third most frequent pathogen of bacterial meningitis in the adult population, following *S. pneumoniae* and *N. meningitidis*, and accounting for 4%–16.5% of cases [1–6].

Studies of LM CNS infections are reported from Europe, USA, Australia, and Asia. In Central and Eastern European countries, reports are scarce and limited to case studies. The aim of this study was to describe the predisposing factors, clinical and laboratory features, treatment, complications, and outcomes for LM meningitis in adults, in a medical center which is representative of conditions in Central/Eastern European countries.

## 2. Materials and Methods

A prospective observational study of adult patients with acute bacterial meningitis (ABM) was carried out at the Infectious Diseases Department of Na Bulovce Hospital in Prague from 1997 to 2012. The department is a tertiary care facility with a catchment area of about 1.7 million people (about one-sixth of the total Czech population) and a major centre for treatment of CNS infections in the Czech Republic. Patients were ≥16 years old and had acute bacterial meningitis with a compatible clinical symptomatology. Diagnosis of LM meningitis was confirmed when three out of four criteria were positive: cerebrospinal fluid (CSF) pleocytosis ≥100 leukocytes/*μ*L, protein concentration >1 g/L, CSF/serum glucose concentration ratio <0.5, and a CSF culture or polymerase chain reaction (PCR) or blood culture positive for LM.

Demographic, clinical, and laboratory data, as well as treatment, complications, and clinical outcomes, were collected prospectively. Elderly patients were defined as those ≥65 years of age. Hematologic malignancy, immunosuppressive therapy (cytostatic drugs, long-term corticosteroids), asplenia, and HIV infection were considered as severe immunosuppression. Diabetes mellitus, controlled with oral antidiabetics or insulin, alcoholic liver disease, chronic renal disease, and autoimmune disease, were considered as other immunosuppressive comorbidities. Arterial hypertension was defined as taking chronic antihypertensive medication or an arterial blood pressure ≥145/95 mmHg without medication. Time to treatment was counted from the onset of meningitis symptoms to the initiation of antibiotic treatment. Mental status was scored using the Glasgow Coma Scale (GCS).

Clinical outcomes were classified immediately in nonsurvivors, and two months after discharge in survivors, using the Glasgow Outcome Scale (GOS) score: 1—death, 2—vegetative state, 3—severe disability (the patient is unable to live independently but can follow commands), 4—moderate disability (the patient can live independently but is unable to return to work or school), and 5—mild or no disability [7].

## 3. Results

Over a sixteen-year period, thirty-one patients with LM meningitis were hospitalized, which was 7% of a total of 440 adult ABM patients. LM was the third most common laboratory confirmed etiological agent in acute ABM in adults after *S. pneumoniae *and *N. meningitidis* causing 30% and 23% of cases, respectively. In total, the etiological agent of ABM was identified in 78% of adult cases.

Nine patients had severe immunosuppression, and ten had other immunosuppressive comorbidity (Table 1). Of the 12 remaining patients, seven were elderly, seven had arterial hypertension, and one had vascular CNS disease. In total, arterial hypertension was found out in 14 patients (45%) with LM meningitis and in 71 out of 409 patients with ABM of other etiology (17%; *P* < 0.001). Only the youngest patient (26 years) with LM meningitis had no predisposing factor.

The majority of patients had typical symptoms of acute bacterial meningitis. At least two out of four typical meningitis symptoms, that is, headache, fever, meningism, and altered mental status, were present in 30 patients (97%).

The median count of CSF leukocytes was 680/*μ*L (polymorphonuclears 346/*μ*L, lymphocytes 100/*μ*L), the median protein level was 2.6 g/L, and CSF/serum glucose ratio was 0.28. Mononuclear cell predominance (more than 50% of cell count) was present in 19% of diagnostic CSF samples. LM was identified by CSF culture in 25 of 30 patients examined (83%) and by blood culture in 13 of 24 patients (54%). Both CSF and blood cultures were positive in eight patients. In one patient, LM was isolated in an autopsied meningeal tissue sample. Antibiotic sensitivity testing of all the isolates did not find any atypical sensitivity or resistance. A CSF Gram stain detected gram-positive rods only in five samples of 16 examined (31%).

Patients were treated using the standard protocol for ABM, that is, antibiotics, dexamethasone, and complex intensive care, including vital functions support. In the majority of cases, the initial antibiotic was cefotaxime or ceftriaxone, in combination with ampicillin when a LM etiology was suspected. In total, 30 patients (97%) were treated with ampicillin (3 g q 6 h, body weight ≤80 kg and 4 g q 6 h, body weight >80 kg), 25 patients in combination with gentamicin 240 mg q 24 h for 7–14 days and five in combination with cotrimoxazole 960 mg q 12 h for 21 days. Dexamethasone was given to 27 patients; four patients referred from other facilities after 24–48 hours of initial antibiotic treatment were not given corticosteroids.

Complications developed in 16 patients (52%), and eight of them had both neurological and systemic complications. The most frequent neurological complications were cerebrovascular events (bleeding or ischaemia) in four patients and severe brain edema in six patients. The most frequent systemic complications were acute renal failure in eight patients and secondary sepsis in four patients. Four patients (13%) died; three of them died of neurological causes (two of intracerebral hemorrhage, one of severe brain edema with herniation), and one patient died of secondary sepsis resulting in multiple organ failure. Nine patients (29%) survived with sequelae: one with severe and eight with moderate disability. Eighteen patients (58%) were cured with mild or no disability. 

## 4. Discussion

From 1997 to 2005, 15–23 cases of invasive LM infection have been reported per year in the Czech Republic, with an incidence of about 0.2 per 100,000 inhabitants [8]. Approximately, 40% were perinatal cases and 60% nonperinatal, mainly meningitis. Between 2006 and 2007, we faced an outbreak of LM infections caused by a technological failure in a cheese manufacture, with a total of 75 cases, including 13 perinatal infections. In the years following, the incidence rate returned to its pre-epidemic figures.

Our study did not find any healthcare-associated case of LM infection, although it has been reported in 3%–30% of cases [4, 5, 9–13]. It is difficult to ascertain the healthcare-associated origin of LM infection, due to the long incubation period, which often exceeds 30 days [14].

It is a known fact that LM causes meningitis predominantly in immunocompromised and elderly persons. Our findings are in accordance: sixty-one percent of our patients had immunocompromising comorbidity, and the majority of the remainder was elderly. In our view, the decline of immune functions associated with ageing does not satisfactorily explain the higher incidence of LM meningitis in the elderly. Additional predisposing factors are likely to be important in the pathogenesis of the disease. Of twelve patients lacking immunosuppressive comorbidity, seven suffered from arterial hypertension and one from vascular CNS disease. Regardless of other comorbidity, arterial hypertension was significantly more common in patients with LM meningitis compared to patients with other ABM (unpublished data). This led us to hypothesize that arterial hypertension could impair the integrity and function of the blood/brain barrier and thus enable the invasion of LM into the subarachnoid space and/or brain tissue. We did not find similar findings in the literature; thus, it would be interesting to focus on the frequency of arterial hypertension in other case series.

The clinical presentation of LM meningitis did not differ from ABM caused by other bacteria. Sixty-eight percent of patients presented with the typical triad of fever, neck stiffness, and altered mental status, compared to 64% of patients with ABM of other bacterial etiologies [15]. Brouwer et al. reported the typical triad of symptoms in 43% of LM meningitis patients [2] and in 44% of all community-acquired ABM patients [16]. The lower prevalence of neck stiffness is also in agreement with previous reports [2, 4, 17, 18]. Majority of our patients had an acute disease onset with time to diagnosis and treatment less than 48 hours.

We observed only mild CSF differences between LM meningitis and meningitis of other bacterial etiologies: lower median count of CSF leukocytes and protein level (680 versus 2,560 cells/*μ*L, 2.6 versus 3.9 g/L, resp.) and higher CSF/serum glucose ratio (0.28 versus 0.13) [3]. We were unable to confirm the absence of hypoglycorrhachia, which has previously been reported [4, 17, 18]. Concordant with other studies was the low sensitivity of the CSF Gram stain [4].

There are no prospective controlled trials on the most efficacious antibiotic treatment of LM meningitis. LM strains isolated from patients are susceptible to a broad range of antibiotics in vitro but only a few antibiotics, namely, aminoglycosides, cotrimoxazole, vancomycin, and the newer quinolones are bactericidal. Despite betalactam antibiotics demonstrate delayed in vitro bactericidal activity at levels obtainable in the CSF, ampicillin or amoxicillin remains the current best practice [19]. Based on synergy in vitro and in animal models, most authorities recommend combination with gentamicin in LM meningitis. All but one of our patients was treated with ampicillin, although it was delayed in 19 patients by a median time of 11 hours, mostly in those patients who were referred after an initial treatment with cefotaxime or ceftriaxone. Treatment with cotrimoxazole as a monotherapy is recommended for patients allergic to penicillins. Cotrimoxazole is bactericidal in vitro on extracellular listeria but its activity on intracellular bacteria is not superior to ampicillin [19]. In a study of Merle-Melet et al. the combination of cotrimoxazole plus ampicillin was associated with lower failure rate and fewer neurologic sequelae than ampicillin with gentamicin. The same study found out failure of ampicillin and gentamicin in 57% of patients [20]. In majority of our patients, we observed a favorable response to the combination of ampicillin plus gentamicin. The therapeutic value of vancomycin is controversial; it can accumulate in host cells but fails to reach the intracellular compartment where listeria multiply [19]. In vitro and animal experiments showed good activity of levofloxacin and moxifloxacin. They are promising agents but the clinical experience is still too limited.

A case-fatality ratio of 13% in our study was less than 17%–61% as reported by other authors [2, 9–13]. Gerner-Smidt et al. ascertained that factors predisposing to LM infection were also associated with a higher case-fatality ratio in patients less than 70 years of age, which was not observed in patients above this age [10]. Fernández Guerrero et al. concluded that fatal outcome of listeriosis mainly depended on the severity of the underlying disease with haematological neoplasia being significantly associated with the risk of death [9]. Three of our nonsurvivors were 32, 52, and 52 years old, and all of them had predisposing internal comorbidities, however no neoplasia: HIV infection, alcoholic liver cirrhosis, and cirrhosis combined with diabetes. The fourth nonsurvivor was 72 years old, and his only comorbidity was arterial hypertension.

The main limitation of the study was the small sample size, which relates to the fact that LM meningitis is a low-frequent disease, and the study was single center. The small number of patients also precluded any meaningful statistical analysis.

## 5. Conclusion

Our findings showed that LM, despite not being commonplace, is the third most common etiological agent of acute ABM in adults. It is mainly found in immunocompromised and elderly persons. Based on our results, arterial hypertension could be considered as another important predisposing factor. Clinical symptoms and CSF findings in LM meningitis did not differ from ABM of other bacterial etiology, with the exception of CSF leukocyte count, which tended to be lower. Ampicillin showed good clinical and bacteriological efficacy and should be included in the initial antibiotic treatment of acute bacterial meningitis in patients with known predisposing factors for LM infection.

## Figures and Tables

**Table 1:** Demographic, clinical, and laboratory characteristics of adults with *L. monocytogenes* meningitis.

Variable	Patients (*n* = 31)
Age [years]^1^	63 (26–80)
Elderly ≥65 years	15/31 (48)
Gender	
Female	14/31 (45)
Male	17/31 (55)
Severe immunosuppression	9/31 (29)
Haematologic malignancy	5
Long-term corticosteroids	3
HIV infection	1
Other immunosuppressive comorbidity	10/31 (32)
Diabetes mellitus	5
Alcoholic liver disease	3
Autoimmune disease	2
Arterial hypertension^2^	14/31 (45)
Time to treatment [hours]^1^	48 (12–96)
≤48 hours	26/31 (84)
Symptoms at presentation^3^	
Fever	31/31 (100)
Altered mental status	27/31 (87)
Headache	19/23 (83)
Meningism	22/31 (71)
Vomiting	6/23 (26)
Seizures	4/26 (15)
Fever, meningism, altered mental status	21/31 (68)
GCS score^1^	11 (3–15)
≤8	3/31 (10)
CSF findings^1^	
CSF leukocytes [1/*μ*L]	680 (133–6,666)
CSF protein [g/L]	2.6 (0.6–7.6)
CSF/serum glucose ratio	0.28 (0.02–0.53)

Data are presented as no./total no. (%).

^1^Median range is used for continuous variables.

^2^Some patients had multiple comorbidities.

^3^Anamnestic data were not complete in eight patients.
